# Die Kaltplasma‐Technologie in der Behandlung von Menschen mit chronischen Wunden

**DOI:** 10.1111/ddg.70053

**Published:** 2026-02-05

**Authors:** Sander Bekeschus, Lars Boeckmann, Alexander Thiem, Steffen Emmert

**Affiliations:** ^1^ Klinik und Poliklinik für Dermatologie Venerologie und Allergologie Universitätsmedizin Rostock Rostock Deutschland; ^2^ ZIK plasmatis Leibniz‐Institut für Plasmaforschung und Technologie (INP) Greifswald Deutschland

**Keywords:** Plasmamedizin, reaktive Sauerstoff‐ und Stickstoffspezies, Ulzera, Wundheilungsstörungen, plasma medicine, reactive oxygen and nitrogen species, ulcers, wound healing disorders

## Abstract

Chronische Wunden stellen eine erhebliche Belastung sowohl für Patienten als auch für Gesundheitssysteme dar. Die Kaltplasma‐Technologie hat sich bei der Behandlung von Menschen mit chronischen Wunden in den letzten Jahren zunehmend etabliert. Mittlerweile sind verschiedene Kaltplasma‐Geräte verfügbar. Allen gemein ist, dass sie körperwarme Plasmen sowie reaktive Sauerstoff‐ und Stickstoffspezies (ROS/RNS) generieren. Diese vermitteln zum einen antimikrobielle Effekte, aber erhöhen auch die Hautdurchblutung und wirken zellproliferativ. Damit liegen der Kaltplasma‐vermittelten, beschleunigten Wundheilung verschiedene Wirkmechanismen, die in einer Behandlung synergistisch wirken, zugrunde. Die seit 2022 veröffentlichte AWMF‐S2K‐Leitlinie zum rationalen therapeutischen Einsatz von kaltem physikalischem Plasma empfiehlt folgerichtig die Wundbehandlung mittels Kaltplasma‐Technologie. Dieser Beitrag gibt einen Überblick über die klinische Evidenz, mögliche Wirkmechanismen sowie Sicherheitsaspekte bei der therapeutischen Anwendung der Kaltplasma‐Technologie bei chronischen Wunden.

## EINLEITUNG

Von einer gestörten Wundheilung sind in der westlichen Welt Millionen von Menschen betroffen. Allein in Deutschland leiden annähernd 2,7 Millionen Menschen an chronischen Wunden, wobei die Prävalenz und Inzidenz bei Personen um beziehungsweise nach dem 70. Lebensjahr zunimmt.^1^

Chronische Wunden sind Wunden, die nach einem längeren Zeitraum trotz sachgerechter Behandlung keine wesentlichen Heilungstendenzen zeigen und daher über den normalen Zeitrahmen der Wundheilung von sechs Wochen hinaus bestehen bleiben. Wunden unterscheiden sich in Alter und Grad der Chronifizierung.^2^, ^3^, ^4^ Im Unterschied zu akuten Wunden, deren Heilungsverlauf primär durch äußere Einflüsse geprägt ist und in der Regel innerhalb weniger Wochen abgeschlossen ist, entstehen chronische Wunden meist in Folge von Grunderkrankungen wie venöser Insuffizienz oder arterieller Verschlusskrankheit, Diabetes mellitus oder lokal durch lang anhaltenden Druck und/oder Scherkräfte (Dekubitalulzera).^5^, ^6^

Der Wundheilungsprozess ist durch vier kontinuierliche, sich überlappende und präzise programmierte Phasen charakterisiert: Hämostase, Entzündung, Proliferation und Remodellierung.^7^ Diese lineare Abfolge der einzelnen Wundheilungsphasen ist bei chronischen Wunden gestört. Dabei können sich verschiedene Bereiche der Wunde in unterschiedlichen Wundheilungsphasen befinden, wobei vor allem eine chronische Entzündung und/oder eine gestörte Neovaskularisation/Kapillarzirkulation (verminderte Nährstoffzufuhr) charakteristisch für chronische Wunden sind.^4^, ^8^

Viele unterschiedliche Zelltypen sind an der normalen Wundheilung beteiligt. Beim Wundverschluss sind Fibroblasten (und Keratinozyten) essenziell für die Ablagerung der extrazellulären Matrix und den Gewebsumbau. Neutrophile Granulozyten spielen eine zentrale Rolle in der frühen Entzündungsphase. Sie wandern unmittelbar nach einer Gewebeschädigung in das Wundgebiet ein, um eingedrungene Mikroorganismen und Zelltrümmer zu beseitigen. Durch Phagozytose, die Freisetzung reaktiver Sauerstoffspezies (ROS) und antimikrobieller Enzyme tragen sie wesentlich zur Infektabwehr und zur Vorbereitung der anschließenden Regenerationsphase bei.^9^ Kommt es jedoch zu einer bakteriellen Infektion, werden neutrophile Granulozyten in übermäßiger Zahl rekrutiert und bleiben dauerhaft aktiviert.^10^ Sie setzen dabei entzündungsfördernde Zytokine, proteolytische Enzyme und sogenannte *Neutrophil Extracellular Traps* (NETs) frei, um die Erreger zu bekämpfen. Auf diese Weise entsteht ein Teufelskreis: Die persistierende bakterielle Besiedlung hält die Neutrophilenaktivität aufrecht, deren freigesetzte Enzyme wiederum das umliegende Gewebe schädigen und die Wundheilung weiter verzögern. Dieser chronisch‐entzündliche Zustand verhindert die Wundheilung und trägt damit zur Aufrechterhaltung einer chronischen Wunde bei. Diesen „Teufelskreis“ zu unterbrechen bleibt eine Herausforderung und sollte das Ziel innovativer Therapieansätze sein.^11^ Einen vielversprechenden innovativen Therapieansatz zur Förderung der Heilung chronischer Wunden stellt hierbei die Kaltplasma‐Behandlung dar.

### Kaltplasma‐Technologie

Der Begriff Plasma stammt aus dem Griechischen und bedeutet „etwas Geformtes“. Plasma wird gemeinhin in der Medizin mit Blutplasma und in der Biologie mit Zytoplasma assoziiert. Das Wort bezeichnet jedoch auch das physikalische Plasma, das als der vierte Aggregatzustand beschrieben wird. So wird angenommen, dass der Großteil der sichtbaren Materie im Universum im Plasmazustand vorliegt. Auf der Erde tritt Plasma beispielsweise in Form von Blitzentladungen, dem Polarlicht (*Aurora borealis*) oder Feuer (Schweißen) auf. Physikalisches Plasma wird erzeugt, indem Gas so stark energetisiert wird, dass Elektronen von Atomen gelöst werden. Da das resultierende ionisierte Gas (Plasma) geladene Teilchen (Elektronen und Ionen) enthält, ist es leitfähig, während die Nettoladung insgesamt aber elektrisch neutral bleibt. Typische weitere erzeugte Teilchen im Plasma umfassen reaktive atomare und molekulare Spezies, die geladen oder neutral sein können. Zudem entstehen elektrische und magnetische Felder sowie elektromagnetische Strahlung unterschiedlicher Wellenlängen (sichtbar, infrarot, ultraviolett).^12^

Medizinische Plasma‐Applikationen sind seit Langem im Bereich der Elektrochirurgie etabliert, wo Techniken wie die Argonplasmakoagulation (APC) auf gezielte thermische Nekrotisierungen von Gewebe zur Hämostase (Kauterisierung) oder zum Schneiden/Entfernen von Gewebe abzielen. Die technische Verfügbarkeit von Aufbauten zur stabilen und reproduzierbaren Plasma‐Zündung bei niedriger Temperatur unter atmosphärischen Bedingungen eröffnete das neue Feld der Plasmaphysik in der Medizin, der Plasmamedizin. Diese umfasst die direkte Anwendung kalter, physikalischer Plasmen auf oder im menschlichen (beziehungsweise. tierischen) Körper zur Erzielung therapeutischer Effekte. Zugelassene Kaltplasma‐Geräte in Europa entsprechen den Vorgaben der Medizinprodukteverordnung, werden bei annährend Körpertemperatur und Atmosphärendruck betrieben und sind üblicherweise als Medizinprodukte der Klasse IIa, zum Teil. aber auch IIb, eingestuft.^13^, ^14^, ^15^

**ABBILDUNG 1:**
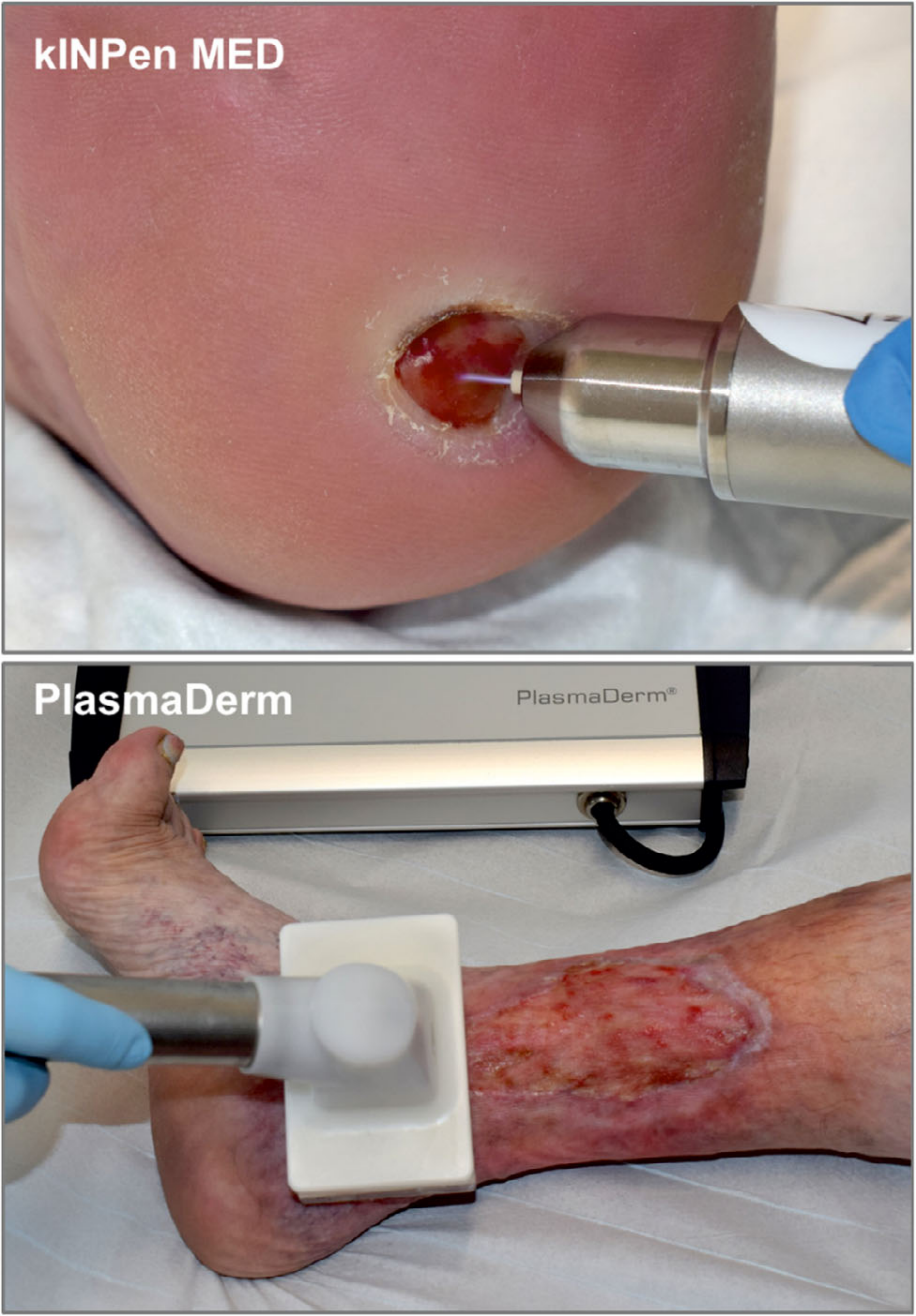
Klinische Anwendung des kINPen^®^ MED und des PlasmaDerm^®^‐Geräts in der unterstützenden Wundbehandlung. Modifiziert nach.^27^

Weltweit wurden die ersten beiden Kaltplasma‐Geräte 2013 in Deutschland als Medizinprodukte zugelassen (Abbildung 1). Inzwischen sind eine Reihe weiterer Geräte hinzugekommen, wobei sie sich teilweise deutlich in der Technologie, wie das Kaltplasma erzeugt wird, unterscheiden. Gemeinhin wird zwischen Plasmajets, Volumen *DBD* (*Dielectric Barrier Discharge*), Oberflächen *DBD* beziehungsweise *Surface‐Micro‐Discharge* (*SMD*) und Plasma‐*Torch* Geräten unterschieden.^16^ Die verschiedenen Technologien bringen unterschiedliche Vor‐ und Nachteile mit sich, die sich jedoch nicht auf die grundsätzlich Plasmawirkung auswirken. So haben Plasmajets ein kleinere Wirkfläche und erlauben dadurch die gezielte Behandlung kleinerer Läsionen und das Eindringen des Kaltplasmas in Kavitäten. Volumen und Oberflächen DBD Plasmaquellen können hingegen größere Flächen abdecken und sind daher für die Kaltplasma‐Anwendung auf größeren Bereichen besser geeignet.

Momentan existieren fünf CE‐gekennzeichnete Kaltplasma‐Geräte auf dem deutschen Markt. Darunter ist der kINPen^®^ MED (neoplas med GmbH), ein Argon‐Plasmajet vor allem für punktgenaue Behandlung chronischer Wunden und Wundtaschen. Darüber hinaus gibt es PlasmaDerm^®^ (CINOGY GmbH), ein DBD‐Gerät, mit welchem größere Flächen behandeln werden können, sowie PlasmaDerm^®^ Dress (CINOGY GmbH), eine plasmaerzeugende Wundauflage welche auch unter dem Verband auf Wunden über längere Zeit wirken kann. Auch der CPT^®^ Patch (Coldplasmatech GmbH) ist eine aktive plasmaerzeugende Wundauflage, welche jedoch nach einmaliger Wundbehandlung nach wenigen Minuten von der Wunde entfernt wird. Ebenfalls eine flächige DBD‐Anwendung, welche an der Umgebungsluft gezündet wird, ist das plasmacare^®^ (terraplasma medical GmbH) Gerät, ein mobiles, Batterie‐betriebenes Handgerät. Das SteriPlas^®^ Gerät (AdTec Ldt.) ist ein Plasma‐Torch Gerät auf Argon‐Basis, welches ebenfalls größere Wundareale versorgen kann. Zu allen Geräten liegen dokumentierte, klinische Erfahrungen vor. Für das ebenfalls CE zertifizierte plasma ONE^®^ (plasma MEDICAL SYSTEMS^®^ GMBH) Gerät ist der klinische Nutzen bei chronischen Wunden nicht wissenschaftlich dokumentiert.

Bisher sind weltweit noch keine klaren, einheitlichen Kriterien für Kaltplasma‐Quellen, die für medizinische Anwendungen eingesetzt werden sollen, definiert. Anforderungen zur Bestimmung der Sicherheit und Wirksamkeit von Geräten zur Erzeugung von kalten Plasmen für medizinischen Anwendungen wurden in Deutschland in einer DIN‐Spezifikation beschrieben (DIN SPEC 91315:2025‐07).^17^ Darüber hinaus gibt es eine AWMF S2k‐Leitlinie „Rationaler therapeutischer Einsatz von kaltem physikalischem Plasma“^18^, an der sich zehn medizinische Fachgesellschaften beteiligt haben und in der Empfehlungen zum rationalen therapeutischen Einsatz von Kaltplasma als Ergänzung zur leitliniengerechten jeweiligen Standardtherapie an Patienten dargelegt sind.

Die Bildung von Kaltplasma soll hier beispielhaft für einen Plasmajet dargestellt werden: Durch Anlegen einer hochfrequenten Wechselspannung an ein Edelgas, etwa Argon, wird Plasma erzeugt. Der Elektronenstrom weist dabei eine hohe kinetische Energie auf und ist „heiß“. Diese schnellen Elektronen ionisieren anschließend Moleküle des Argon‐Arbeitsgases. Im Gegensatz dazu sind Argonmoleküle (Ionen) deutlich massereicher als Elektronen und bewegen sich daher langsamer im elektrischen Feld. Außerdem übertragen schnelle Elektronen ihre kinetische Energie ineffizient auf Ionen, die deshalb kalt bleiben. Da die Ionentemperatur eines Gases dessen Gesamt‐Temperatur bestimmt, enthält das Plasma einerseits hochenergetische Teilchen, zeigt andererseits jedoch nicht den Temperaturanstieg, der sonst mit deren Anwesenheit einhergehen würde: ein sogenanntes Nichtgleichgewichtsplasma. Eine zusätzliche Abkühlung des Plasmas kann durch hohe Arbeitsgas‐Volumenströme erzielt werden. Das Arbeitsgas treibt das Plasma und seine geladenen Argonteilchen in die umgebende Luft, die Sauerstoff und Stickstoff enthält, und erzeugt schließlich reaktive Sauerstoff‐ und Stickstoffspezies (ROS/RNS). Andere Kaltplasma‐Technologien, bei denen atmosphärische Luft als Arbeitsgas verwendet wird, erzeugen ebenfalls ROS/RNS. Dies ist damit eine generelle Eigenschaft kalter atmosphärischer Plasmen. Weitere Details zur Technologie und Plasmazündung verschiedener Geräte‐Konzepte finden sich in Übersichtsarbeiten.^19^, ^20^

### Präklinische Studien

Die antimikrobielle Wirksamkeit von Kaltplasma‐Geräten erstreckt sich auf Viren, Pilze und Bakterien und schließt bspw. die Bakterienspezies *Staphylococcus aureus*,^21^ *Pseudomonas aeruginosa*,^22^ und *Escherichia coli* ein.^23^ Mehr als 100 klinische Isolate von Wundbakterien, darunter auch multiresistente Stämme, wurden von Kaltplasma‐Geräten in vitro erfolgreich eliminiert.^24^ Eine Ausbildung bakterieller Resistenz gegen Kaltplasma‐Behandlung wurde bislang nicht beobachtet.^25^, ^26^

Mehrere Dutzend Wundheilungsstudien in zumeist Nagetiermodellen konnten eine beschleunigte Wundheilung nach Kaltplasma‐Behandlung nachweisen und potenzielle Wirkmechanismen aufzeigen,^27^ von denen eine Auswahl hier dargestellt wird. An dieser Stelle ist es wichtig zu erwähnen, dass, obwohl eine vermehrte Kolonisation oder Infektion durch Bakterien die Wundheilung meist negativ beeinflussen,^28^ deren Eradikation nicht immer mit einer Verbesserung der Heilungsreaktionen verbunden ist.^29^ In verschiedenen Mausmodellen mit nicht‐infizierten Wunden konnte eine verbesserte Wundheilung durch Kaltplasma‐Anwendung gezeigt werden, wodurch der Kaltplasma‐Technologie (neben antiseptischen Eigenschaften) auch direkt regenerative Eigenschaften zugeschrieben werden konnten. In Mäusen mit dorsalen Punch‐Biopsie‐Wunden wurde gesteigerte Angiogenese Makrophagen‐ und Neutrophileninfiltrate, begleitet von erhöhten Konzentrationen von Fibroblasten‐Wachstumsfaktor 2 (FGF2), Monocyte‐Chemoattractant Protein 1 (MCP‐1) und Interleukin‐6 (IL‐6) beobachtet.^30^, ^31^ Studien an Ohrwunden von Mäusen bestätigten und erweiterten diese Befunde und zeigten eine Kollagenfaser‐ und Keratinproduktion, Granulationsgewebe‐Entstehung, sowie antioxidative (*Nuclear Factor Erythroid 2‐Related Factor 2*, Nrf2) und proliferative (*cellular tumor antigen p53*, p53) Antworten im Gewebe.^32^, ^33^, ^34^ Letztere Studie zeigte zudem, dass sich für mehrere Parameter (Re‐Epithelialisierung; IL‐1β, IL‐6, (*tumor growth factor*) TGF‐β, Nrf2, (*heme oxygenase 1*) HMOX1, (*NAD(P)H:Quinon‐Oxidoreduktase 1*) NQO1, (*superoxide dismutase 1*) SOD1, (*catalase*) CAT, (*keratinocyte growth factor*) KGF) Unterschiede zwischen kurzen (3 s) und längeren (20 s) Expositionszeiten ergaben. Dies deutet darauf hin, dass die differentielle Redox‐Regulation von der insgesamt in die Wunde deponierten Menge an ROS/RNS abhängt. Zusätzlich erhöhte die Kaltplasma‐Behandlung die Gewebeoxygenierung der Wunde in oberflächlichen und tiefen Schichten.^35^

Neben den in diesen Studien genutzten CE zertifizierten, zugelassenen Kaltplasma‐Geräten wird auch mit experimentellen Kaltplasma‐Quellen eine verbesserte Wundheilung in Tiermodellen beobachtet.^36^ Dabei konnte die wundheilungsfördernden Eigenschaften des Kaltplasmas verbessert werden, wenn Betriebsparameter, wie beispielsweise das Arbeitsgas,^37^, ^38^, ^39^ oder die Plasma‐Anregungsfrequenz,^40^ Anzahl der Kaltplasma‐Behandlungen,^41^ sowie die Expositionszeit,^42^, ^43^ verändert werden.^44^ Untersuchungen in diabetischen Tieren bestätigten die Wundheilungsverbesserung mittels Kaltplasma‐Behandlung durch Modifikation von Signaltransduktionsprozessen im Hippo‐ und Nrf2‐Weg.^45^, ^46^ Diese Erkenntnisse sind dahingehend wichtig, da diabetische Patienten besonders anfällig für chronische Wundheilungsstörungen sind.

Präklinische Untersuchungen in vivo zeigen, dass die Kaltplasma‐Behandlung die Wundheilung auch in nicht‐infizierten Wunden fördert.

### Pathomechanismen der Kaltplasma‐unterstützten Wundheilung

Lange wurde vermutet, die antimikrobielle Aktivität der Kaltplasma‐Behandlung sei primär für die verbesserte Heilung verantwortlich. Studien an nicht experimentell‐infizierten Tierwunden sowie Patienten, die eine Kaltplasma‐Behandlung in Kombination mit Antiseptika erhielten,^47^ deuten jedoch auf zusätzliche Wirkmechanismen hin, die unabhängig von der Inaktivierung von Mikroorganismen sind.^47^ Faktoren, die möglicherweise zur Kaltplasma‐gestützten Wundheilung beitragen, sind die, etwa mit Hyperspektral‐Imaging erfassten und unmittelbar nach der Kaltplasma‐Behandlung messbaren, Gewebereaktionen. So wurde nach Kaltplasma‐Behandlung von Wunden an Ohren von Mäusen eine gesteigerte oberflächliche und tiefe Mikrozirkulation sowie Gewebeoxygenierung gemessen.^48^ Ähnliche Befunde wurden an Kaltplasma‐behandelten Patientenwunden,^49^ und an intakter menschlicher Haut berichtet.^50^, ^51^, ^52^

Mehrere Mechanismen könnten diese Effekte erklären: Plasmajets wie der kINPen^®^ MED generieren hohe Mengen an Stickstoffmonoxid (NO),^53^, ^54^ das bekanntlich arterielle Vasodilatation bewirkt und damit den Blutfluss erhöht. Dies könnte durch die intrinsische Wärmeenergie des Plasmas (in der Größenordnung von Körpertemperatur beziehungsweise leicht darüber, ca. 37–40 °C) noch verstärkt werden, da eine Erwärmung des behandelten Gewebes (Haut ∼30 °C) die endogene NO‐Produktion anregen kann,^55^ und so den Blutfluss weiter verstärkt. Eine gesteigerte Mikrozirkulation verbessert den Transport von Nährstoffen, die Transmigration neuer, nicht voraktivierter Leukozyten und schließlich auch die Sauerstoffversorgung, wodurch Hypoxie – einem Schlüsselfaktor für Ulzerationen – entgegengewirkt wird.^56^ Hypoxie wird zusätzlich durch gesteigerte Angiogenese kompensiert, wie in mehreren Tiermodellen nach Kaltplasma‐Behandlung beobachtet, die eine anhaltend erhöhte Vaskularisierung und Gewebeoxygenierung zeigten.^30^, ^34^, ^57^ Dies deutet daraufhin, dass die Kaltplasma‐Behandlung in die Redox‐Kontrolle von Zellen und Geweben eingreift. In vivo wurde eine ausgeprägte Aktivierung des antioxidativen nukleären Transkriptionsfaktors Nrf2 festgestellt, der zudem bereits als vielversprechendes pharmakologisches Ziel zur Förderung der Wundheilung beschrieben wurde.^58^ In intakter muriner Haut führte Kaltplasma‐Behandlung zu erhöhter Zellproliferation sowie zu gesteigerter Expression von Nrf2 und Katalase.^59^

Jedoch bilden in vivo‐Modelle die klinische Situation nur begrenzt ab, da Grunderkrankungen oder ausgeprägte Infektionen, die in der Klinik zur Chronifizierung von Wunden beitragen, in diesen nicht berücksichtigt sind. So ist in Tiermodellen die Wundheilung mitunter verzögert, bleibt aber funktional; bei Patienten ist sie häufig vollständig zum Stillstand gekommen. Die entscheidende Frage lautet daher oftmals nicht allein, wie Wundheilung gefördert, sondern wie sie überhaupt wieder in Gang gesetzt wird – oft beschrieben als die Umwandlung einer chronischen in eine akute Wunde.

Als Ursachen für den persistierenden Zustand chronischer Wunden werden multifaktorielle Prozesse wie Hypoxie, Infektion und seneszente Zellen angenommen.^60^ Insbesondere seneszente Fibroblasten,^61^ und Neutrophile,^62^ werden dabei mit beeinträchtigter Heilung assoziiert. Die Induktion von Apoptose durch Kaltplasma‐bedingten oxidativen Distress,^63^ in moderatem Ausmaß, könnte ein weiterer Wirkmechanismus von Kaltplasma sein und stellt eine testbare Hypothese für die klinische Kaltplasma‐Wundforschung dar. Viele Wundtherapien zielen allgemein auf die Verringerung entzündlicher Prozesse. Angesichts der Vielzahl beteiligter Zelltypen, der Dutzenden von Chemokinen und Zytokinen und deren komplexer Wechselwirkungen in den unterschiedlichen Heilungsphasen ist es nachvollziehbar, dass bislang keine einfachen Lösungen zur optimalen Regulierung der Wundheilung identifiziert werden konnten.^64^

Chronische Wunden sind wiederholt Mikroverletzungen und Blutungen ausgesetzt, insbesondere während Débridement‐Prozeduren zur Entfernung nekrotischen Materials. In Leberverletzungsmodellen bei Mäusen wurde gezeigt; dass Kaltplasma‐Behandlungen hämostatisch wirken und diese Koagulation abhängig von eine Thrombozytenaktivierung war.^65^, ^66^ Diese Thrombozytenaktivierung initiiert eine Kaskade wundheilungsfördernder Ereignisse, unter anderem die Sekretion von Faktoren wie dem thrombozyten‐abgeleiteten Wachstumsfaktor (PDGF) und dem epidermalen Wachstumsfaktor (EGF),^67^ die eingehend als biologische Therapien zur Förderung der Wundheilung in klinischen Studien untersucht wurden.^68^ Passend dazu hat sich autologes Thrombozytengel als Therapie für Wundheilung, Hämostase und lokale Infektionskontrolle etabliert.^69^ Auch wurde vorgeschlagen, elektrische Pulsstimulation könne Thrombozyten in situ aktivieren.^70^ Die Kaltplasma‐Behandlung könnte einen ähnlichen Effekt ausüben.

### Datenlage beim Menschen

In den ersten randomisierten, prospektiven klinischen Studien zur Wundheilung auf dem Gebiet der Plasmamedizin wurde durch eine Behandlung mit dem SteriPlas^®^‐Gerät (AdTec Ltd.) die mikrobielle Belastung chronischer Wunden signifikant bei 36 respektive 24 Patienten reduziert.^71^, ^72^ Auch in einer Nachfolgestudie mit 70 Patienten wurde eine signifikante bakterielle Reduktion festgestellt.^73^ Das SteriPlas^®^‐System wurde zudem erfolgreich zur Förderung der Heilung von Hauttransplantat‐Entnahmestellen in einer randomisierten, Placebo‐kontrollierten Studie mit 40 Patienten eingesetzt.^74^ Die gute Wirksamkeit des Systems zur Unterstützung der Wundheilung konnte in weiteren randomisierten Studien mit 37 beziehungsweise 15 Patienten bestätigt werden.^75^, ^76^ Auch das Gerät PlasmaDerm^®^ (Cinogy GmbH) erzielte in einer weiteren randomisiert‐prospektiven Studie mit 14 Patienten eine signifikante Verringerung der bakteriellen Last und der Wundgröße.^77^ Dasselbe Gerät eignete sich in einer kleinen Studie mit sechs Patienten auch zur Behandlung chirurgischer Wunden,^78^ und steigerte die Durchblutung von Wunden.^79^

Für den Plasmajet kINPen^®^ (neoplas med GmbH) liegen mehrere Berichte zu klinisch relevanten wundspezifischen Effekten vor. Bei experimentell durch Laser induzierten akuten Wunden an den Unterarmen von fünf Versuchspersonen förderte die kINPen®‐Kaltplasma‐Behandlung objektive Heilungsreaktionen,^80^ ohne langfristige (5‐Jahres follow‐up,^81^) unerwünschte Effekte wie Narbenbildung zu verursachen.^82^ Eine ähnliche Untersuchung an zwölf Patienten bestätigte diese Ergebnisse und beobachteten zudem weder eine Zunahme noch Abnahme der Melaninproduktion.^83^ Eine signifikant verbesserte Heilung zeigte sich ebenfalls bei vakuuminduzierten Wunden an Versuchspersonen nach kINPen^®^‐Kaltplasma‐Behandlung.^84^ Eine kleine Studie an vier Patienten mit Laser‐induzierten, akuten Wunden am Unterarm deutete darauf hin, dass kINPen^®^‐Plasma auch in diesem Kontext heilungsfördernd eingesetzt werden könnte.^85^ Die Kaltplasma‐Behandlung chronischer Unterschenkelulzera mit dem kINPen^®^ führte in einer Fallserie von 16 Patienten zu einer signifikant höheren mikrobiellen Reduktion.^86^ In einer größeren Studie mit 34 Patienten mit chronischen Unterschenkelulzera konnte gezeigt werden, dass die Kaltplasma‐Behandlung mit dem kINPen^®^ die mikrobielle Besiedlung in den Wunden signifikant reduzierte.^87^ Interessanterweise veränderten Antiseptika, nicht jedoch die Kaltplasma‐Behandlung das Wundmikrobiom, was darauf hindeutet, dass Plasma unabhängig von unterschiedlichen Bakterienspezies vergleichbare Effekte erzielt. Kürzlich bestätigte eine randomisierte prospektive klinische Studie an Patienten mit diabetischen Fußulzera erneut die wundheilungsfördernden Eigenschaften der kINPen^®^‐Kaltplasma‐Behandlung.^47^ Auffällig ist, dass die Kaltplasma‐Behandlung zusätzlich zu bereits eingesetzten Wundantiseptika erfolgte, was nahelegt, dass der Wirkmechanismus der Kaltplasma‐Behandlung unabhängig oder zumindest weniger abhängig von ihrer antimikrobiellen Wirksamkeit ist (siehe oben). Dies wurde kürzlich in einer weiteren Multi‐Center‐Studie mit 78 Patienten bestätigt.^88^

Neueste Konzepte in der Plasmatechnologie nutzen beispielsweise Einmal‐Pflaster, in die plasmaphysikalische Komponenten integriert sind und welche auf den Wunden verbleiben können, wodurch Kaltplasma mehrfach appliziert werden kann, ohne dass zeitaufwändige Verbandswechsel durch medizinisches Personal erforderlich sind. Erst kürzlich wurde eine erste kontrollierte Pilotstudie zur Verträglichkeit und Wirkung eines neuartigen (DBD)‐basierten Wundverbands PlasmaDerm^®^ Dress publiziert.^89^ Zehn Patienten mit Spalthauttransplantaten (akuten Wunden) wurden eingeschlossen. Die Kaltplasma‐Behandlung der Transplantat‐Entnahmestellen verbesserte signifikant die Durchblutung, einhergehend mit vermindertem Wundschmerz und guter Verträglichkeit. Vermindertes Schmerzempfinden bei gleichzeitig guter Verträglichkeit konnte darüber hinaus in der Anwendung des PlasmaDerm^®^ Dress an chronischen Wunden bestätigt werden.^90^ Dieses PlasmaDerm^®^ Dress‐System (Cinogy GmbH) strebt derzeit die Zertifizierung an. Die ColdPlasmaTech GmbH (Greifswald, Deutschland), arbeitet ebenfalls an entsprechenden Technologien und konnte in einer randomisierten klinischen Studie eine gute Verträglichkeit und Wirksamkeit nachweisen.^91^ Der Unterschied zum PlasmaDerm^®^ Dress liegt beim CPT^®^ Patch jedoch darin, dass dieses großflächige Wundpflaster nur für wenige Minuten für die Dauer der Kaltplasma‐Behandlung auf der Wunde verbleibt. Die Wunden werden anschließend nach jeweiligem Standard versorgt bis beispielsweise beim nächsten Verbandswechsel und Wundreinigung ein neues, steriles CPT^®^ Patch aufgelegt und angewendet werden kann. Schließlich wird seit einigen Jahren an der Zulassung eines Batterie‐betriebenen Kaltplasma‐Gerätes geforscht, welches in einer randomisierten Studie mit 70 Patienten mit chronischen Wunden vielversprechende Ergebnisse erzielte.^92^ Außerdem existieren weiterhin eine Reihe von Studien und Fallberichten mit nicht‐zugelassenen Geräten (Tabelle 1), auf die hier nicht näher eingegangen wird.

**TABELLE 1:** Veröffentlichungen zu Anwendung von zugelassenen oder experimentellen Kaltplasma‐Technologien bei Wunden des Menschen. Veröffentlichungen sind nach Erscheinungsjahr der Publikation sortiert. random. = randomisiert.

Krankheitsbild	Anzahl an Patienten / Probanden	Kaltplasma‐Technologie	Resultat	Referenzen
Chronische Wunden	36 (random.)	SteriPlas®	Reduzierte Bakterienlast	Isbary et al.^71^
Chronische Wunden	24 (random.)	SteriPlas®	Reduzierte Bakterienlast	Isbary et al.^72^
Experimentelle Wunden (Laser)	5 (Probanden)	kINPen® MED	Verbesserte Wundheilung	Metelmann et al.^80^
Transplant‐Wunden	40 (random.)	SteriPlas®	Verbesserte Wundheilung	Heinlin et al.^74^
Chronische Wunden	70 (random.)	SteriPlas®	Verbesserte Wundheilung	Isbary et al.^73^
Experimentelle Wunden (Laser)	5 (Probanden)	kINPen® MED	Reduzierte Entzündung	Metelmann et al.^82^
Experimentelle Wunden (Vakuum)	6 (Probanden)	kINPen® MED	Verbesserte Wundheilung	Vandersee et al.^84^
Chronische Wunden	14 (random.)	PlasmaDerm®	Reduzierte Bakterienlast	Brehmer et al.^77^
Chronische Wunden	34 (Fallsammlung)	kINPen® MED	Reduzierte Bakterienlast	Klebes et al.^87^
Chronische Wunden	16 (Fallsammlung)	kINPen® MED	Reduzierte Bakterienlast	Ulrich et al.^86^
Chronische Wunden	50 (random.)	Experimentell (Microbeam)	Reduzierte Bakterienlast, verbesserte Wundheilung	Chuangsuwanich^108^
Brandwunden (2. Grad)	1 (Fallstudie)	Experimentell (Helium‐Jet)	Reduzierte Entzündung	Betancourt‐Angeles et al.^109^
Transplant‐Wunden mit Wundheilungsstörung	4 (Fallsammlung)	kINPen® MED	Reduzierte Entzündung, verbesserte Wundheilung	Hartwig et al.^85^
Chronische Wunden (MKG)	6 (Fallsammlung)	PlasmaDerm®	Verbesserte Wundheilung	Hartwig et al.^78^
Chronische Wunden	4 (Fallsammlung)	Experimentell (Plasma‐Jet)	Erhöhte Wachstumsfaktoren	Naderi et al.^110^
Chronische Wunden	1 (Fallstudie)	Experimentell (Helium‐Jet)	Verbesserte Wundheilung	López‐Callejas et al.^111^
Chronische Wunden	32 (Fallsammlung)	Experimentell (Helium‐DBE)	Verbesserte Wundheilung	González‐Mendoza et al.^112^
Experimentelle Wunden (Laser)	12 (Probanden)	kINPen® MED	Verbesserte Wundheilung	Nishijima et al.^83^
Diverse Wunden	7 (Fallsammlung)	Experimentell (Multi‐electrode DBE)	Verbesserte Wundheilung	Gao et al.^113^
Chronische Wunden	44 (random.)	Experimentell (Helium‐Jet)	Reduzierte Bakterienlast	Amini et al.^114^
Chronische Wunden	44 (random.)	Experimentell (Helium‐Jet)	Reduzierte Bakterienlast, verbesserte Wundheilung	Mirpour et al.^115^
Chronische Wunden	45 (random., Multi‐Center)	kINPen® MED	Verbesserte Wundheilung	Stratmann et al.^47^
Transplant Wunden	10 (random.)	PlasmaDerm® Patch	Verbesserte Durchblutung, reduzierter Schmerz	Van Welzen et al.^89^
Chronische Wunden	37 (random.)	SteriPlas®	Verbesserte Wundheilung	Moelleken et al.^75^
Chronische Wunden	20	PlasmaDerm® Patch	Verbesserte Durchblutung	Jensen et al.^90^
Chronische Wunden	20 (random.)	Experimentell	Verbesserte Wundheilung	Samsavar et al.^116^
Chronische Wunden	20	PlasmaDerm®	Verbesserte Durchblutung	Schleusser et al.^79^
Chronische Wunden	78 (random., Multi‐Center)	kINPen® MED	Verbesserte Wundheilung	Strohal et al.^88^
Chronische Wunden	15 (random.)	SteriPlas®	Verbesserte Wundheilung	Wiegand et al.^76^
Chronische Wunden	20	Experimentell (Plasoma)	Reduzierte Bakterienlast, verbesserte Wundheilung	Lagrand et al.^117^
Chronische Wunden	48 (random.)	CPT® Patch	Verbesserte Wundheilung	Abu Rached et al.^91^
Chronische Wunden	46 (random.)	Experimentell (Plasoma)	Verbesserte Wundheilung	Bakker et al.^118^
Chronische Wunden	40 (Multi‐Center)	PlasmaCare®	Verbesserte Wundheilung	Ligresti et al.^119^
Chronische Wunde	1 (Fallstudie)	Experimentell (Argon‐Jet)	Verbesserte Wundheilung	Bakhtiyari‐Ramezani et al.^120^
Chirurgische Wunden (u.a. Laparoskopie)	50 (random.)	Experimentell (Helium‐Jet)	Verbesserte Wundheilung	Rodríguez‐Méndez et al.^121^
Chronische Wunde	1 (Fallstudie)	Experimentell (Argon‐Jet)	Verbesserte Wundheilung	Akbartehrani et al.^122^
Brandwunde	1 (Fallstudie)	CPT® Patch	Verbesserte Wundheilung	Milewski et al.^123^
Pediatrische Brandwunden	40 (random.)	Experimentell (Helium‐Jet)	Verbesserte Wundheilung	Rodriguez‐Ferreyra et al.^124^
Chronische Wunde	25	Experimentell	Verbesserte Wundheilung, verändertes Mikrobiom	Gao et al.^125^
Chronische Wunde	70 (random.)	PlasmaCare®	Verbesserte Wundheilung	Strohal et al.^92^
Wunden nach chirurgischer Rekonstruktion peripherer Arterien	50	kINPen® MED	Verbesserte Wundheilung und weniger chirurgische Revisionen	Werra et al.^126^

Es gibt überzeugende Evidenz für einzelne Geräte unterschiedlicher Technologien (Jet vs. DBD) zu wundheilungsfördernden Eigenschaften.

Die Wundheilung‐fördernde Effekte konnten für unterschiedliche Arbeitsgase (Argon, Atmosphärenluft) gefunden werden.

### Sicherheitsaspekte der Kaltplasma‐Technologie und –Behandlung

Neuartige Therapien müssen neben vielversprechender klinischer Wirksamkeit auch ein akzeptables Nebenwirkungsprofil aufweisen. In den klinischen Studien (Tabelle 1) wurde berichtet, dass die Kaltplasma‐Behandlung gut toleriert wird, ohne dass schwerwiegende unerwünschte Ereignisse (SAEs, *serious adverse events*) beobachtet wurden. Mehrere Kaltplasma‐Geräte wurden zudem in Bezug auf potentiell mutagene Eigenschaften (durch oxidative DNA Schäden) untersucht. Für das SteriPlas^®^‐Gerät wurde im HGPRT‐Assay (Hypoxanthin‐Guanin‐Phosphoribosyltransferase) nach (*Organization for Economic Co‐operation and Development*) OECD‐Protokoll (OECD *Guideline for the Testing of Chemicals, Number* 476, adapoted July 1997,^93^) keine Genotoxizität festgestellt.^94^ Eine ähnliches Fehlen mutagener Effekte im HGPRT‐Assay wurde auch für den kINPen^®^ gefunden.^95^ Ein weiterer, nach OECD akkreditierter Genotoxizitätsreferenztest, der Cytokinesis‐Block‐Mikronukleus‐Assay (CBMN), identifizierte in kINPen^®^‐Kaltplasma‐behandelten Zellen keine vermehrte Bildung von Mikronuklei im Vergleich zu positiven Kontrollen wie ionisierender Strahlung, UV‐Strahlung und genotoxischen Wirkstoffen.^96^, ^97^ Zur indirekten Untersuchung von Mutagenität wurde bei Mäusen nach Kaltplasma‐Behandlung von Wunden über einen Zeitraum von einem Jahr – das entspricht etwa 60 Menschenjahren – keine Tumorbildung beobachtet.^33^ Die verheilten Wunden zeigten zudem keine Hautauffälligkeiten, wie eine hypertrophe Narbenbildung.

Auch bei Patienten bestätigten ein‐ und fünfjährige Nachuntersuchungen Kaltplasma‐ behandelter Wunden mittels Hyperspektral‐Bildgebung und konfokaler Laser‐Scanning‐Mikroskopie das Fehlen abnormaler Heilungsreaktionen.^81^, ^82^

Weitere Nebenwirkungen wie ausgeprägte Schmerzen oder Unwohlsein während der Kaltplasma‐Behandlung wurden nicht beobachtet.^89^, ^98^, ^99^, ^100^

### Häufigkeit der Kaltplasma‐Behandlung und Tiefenwirkung

In akuten, Laser‐induzierten Wunden von gesunden Probanden verbesserten sich die Heilungsbefunde sowohl bei dreimal täglicher,^89^ dreimal wöchentlicher,^47^, ^101^ als auch einmal wöchentlicher,^75^ Kaltplasma‐Behandlung, was auf ein breites therapeutisches Anwendungsfenster dieser Technologie hindeutet. Dies lässt sich auch aus Daten tierexperimenteller Wunden schlussfolgern, in denen eine kurze (5s) Kaltplasma‐Behandlung im Vergleich zu unbehandelten Wunden zu einer signifikant beschleunigten Wundheilung führte, und eine längere Kaltplasma‐Behandlung (20s) eine ähnlich gute Heilung zeigte. In der AWMF S2k Leitlinie zum Plasmaeinsatz werden grundsätzlich eher wenige Applikationen pro Woche (2–3) empfohlen. Die Behandlungsdauer ist herstellerspezifisch und schwankt zwischen 30 Sekunden und bis zu 60 Sekunden pro Behandlung und cm^2^ Wundfläche. In Studien sind Behandlungszeiträume von zwei bis acht Wochen gut dokumentiert. Zur Frage, ob eine zu lange oder zu oft‐wiederholte Kaltplasma‐Behandlung auch negative Effekte auf die Heilung chronischer Wunden in der Klinik haben könnte, gibt es keine belastbaren publizierten klinischen Daten. Aus eigener Erfahrung über zehn Jahre und der Routineanwendung der Kaltplasma‐Behandlung im Rahmen unserer multimodalen Wundtherapie sind negative Effekte jedoch nicht zu erwarten.

Die Kaltplasma‐Behandlung sollte im Allgemeinen für einige Wochen (2–8) und 1–3‐mal pro Woche für bis zu 60 Sekunden / cm^2^ Wundfläche erfolgen.

Ein weiterer Aspekt der Kaltplasma‐Behandlung ist die Tiefenwirksamkeit, welche jedoch unzureichend verstanden ist. Sehr wahrscheinlich hängt diese auch vom verwendeten Gerät ab; direkte Vergleichsstudien dazu existieren jedoch nicht. In gesunden humanen oralen Mukosa‐Stanzbiopsien wurde beispielsweise nach kINPen^®^ Behandlung ex vivo keine vermehrte Phosphorylierung des Histons H2A.X sowohl direkt unter dem Stratum corneum als auch in tieferen Hautschichten festgestellt.^102^ In der Haut unbehaarter Mäuse, die mit Kaltplasma behandelt wurden, zeigte sich mittels *tape‐stripping‐assay* und massenspektrometrischer Lipidoxidations‐Analyse, dass bereits die ersten drei Schichten, die durch das *tape‐stripping* vom Stratum corneum abgenommen wurden, einen großen Teil der Kaltplasma‐generierten reaktiven Spezies absorbieren.^103^ Da Wunden typischerweise nicht durch das Stratum corneum geschützt sind, sind hier tiefer‐wirkende Effekte der Kaltplasma‐Behandlung vorstellbar, jedoch vermutlich auch auf wenige Dutzend Mikrometer Tiefe begrenzt, wie Daten zu (yes‐associated protein 1/ t*ranscriptional coactivator with PDZ‐binding motif*) YAP/TAZ‐exprimierenden Hautzellen in murinen Wunden nach Kaltplasma‐Behandlung suggerieren.^46^

Benannte Kontraindikationen können je nach Gerätebeschreibung variieren, sind jedoch im Allgemeinen als gering anzusehen. Besondere Beachtung sollten Patienten mit elektrisch leitfähigen Implantaten erfahren sowie Patienten, die in den letzten sechs Monaten eine neu aufgetretene Herzinsuffizienz entwickelten (elektromagnetische Plasmaströme). Eine Schwangerschaft wird grundsätzlich aus Sicherheitsgründen als Kontraindikation benannt.

### Schlussfolgerungen

Kaltplasma‐Technologien zeigen ausgeprägte antimikrobielle und proliferationsfördernde Effekte bei chronischen Wunden des Menschen. Dies ist unabhängig von dem verwendeten Gerät. Mehrere kontrollierte und randomisierte prospektive Multicenterstudien belegten, dass die Hinzunahme der Kaltplasma‐Behandlung zur Standardversorgung die Wundheilung signifikant förderte. Alle Studien bei Menschen beschrieben eine gute Verträglichkeit der Kaltplasma‐Behandlung und keine schweren Nebenwirkungen. Diese Erkenntnisse flossen in die AWMF S2k‐Leitlinie „Rationaler therapeutischer Einsatz von kaltem physikalischem Plasma“ ein.

Kaltplasma wird als zusätzliche Behandlung chronischer Wunden angewendet und nicht als Ersatz der Standardbehandlung von Wunden angesehen.

### Herausforderungen und klinischer Ausblick

Trotz der überzeugenden Evidenz für den unterstützenden Einsatz von Kaltplasma in der Versorgung chronischer Wunden, bestehen aktuell noch zahlreiche offene Fragen. So existieren bisher keine direkten Vergleichsstudien der unterschiedlichen Kaltplasma‐Geräte. Die optimale Expositionsdauer und ‐frequenz der Kaltplasma‐Anwendung bei der Therapie chronischer Wunden ist noch ungeklärt. Außerdem ist die Auswirkung von Infektionsstatus und Wundlokalisation auf den Behandlungserfolg bisher nicht eindeutig definiert.

Als Ergänzung der deutschen AWMF S2k‐Leitlinie ist die Entwicklung einer S3‐Leitlinie notwendig. Internationale Leitlinien sollten erarbeitet und fortlaufend weiterentwickelt werden, um eine effektive klinische Kaltplasma‐Behandlung zu definieren und dadurch auch die damit verbundenen Geräteanforderungen weltweit. Kombinationstherapien stellen einen weiteren vielversprechenden Ansatz zur Verbesserung Kaltplasma‐basierter Wundheilung dar. In Anlehnung an das eingangs beschriebene Konzept der Redoxregulation der Wundheilung,^104^, ^105^, ^106^, könnte es hier z.B. sinnvoll sein, Kaltplasma mit zusätzlicher Sauerstoffapplikation zur Wundtherapie zu koppeln.^107^

## DANKSAGUNG

Open access Veröffentlichung ermöglicht und organisiert durch Projekt DEAL.

## INTERESSENSKONFLIKT

Steffen Emmert und Alexander Thiem erhielten finanzielle und materielle Forschungsunterstützung für Patientenstudien mit Geräten der Cinogy GmbH. Sander Bekeschus und Lars Böckmann erklären, keine Interessenskonflikte zu haben

## [CME Questions / Lernerfolgskontrolle]

1. Was ist die wichtigste Eigenschaft von Kaltplasma im medizinischen Einsatz?
  - Es erzeugt hohe Temperaturen über 100 °C
  - Es ist elektrisch nicht leitfähig
  - Es enthält reaktive Sauerstoff‐ und Stickstoffspezies (ROS/RNS)
  - Es besteht ausschließlich aus Protonen und Neutronen
  - Es verursacht gezielt thermische Nekrosen
2. Welche Wirkung von Kaltplasma wurde in Tierstudien nachgewiesen?
  - Verstärkte Tumorbildung
  - Beschleunigte Angiogenese und Wundheilung
  - Vermehrte Fibrose ohne Heilung
  - Dauerhafte Hautschädigung
  - Suppression der Mikrozirkulation
3. Welche Mikroorganismen wurden durch Kaltplasma in vitro erfolgreich eliminiert?
  - E. coli
  - Pseudomonas aeruginosa
  - Staphylococcus aureus
  - MRSA
  - Alle Antworten sind richtig
4. Für welchen Signaltransduktionsweg bestehen Hinweise für seine Relevanz im Rahmen der Wundheilung im Rahmen der Kaltplasma‐Therapie?
  - Hippo‐ und Nrf2‐Signalweg
  - Insulin‐Signalweg
  - MAPK/ERK‐Signalweg
  - Wnt/β‐Catenin‐Signalweg
  - VEGF‐Signalweg
5. Welche Leitlinie ist für den therapeutischen Einsatz von Kaltplasma in der Behandlung chronischer Wunden an relevantesten?
  - AWMF S1‐Leitlinie „Chronische Wunden“
  - AWMF S2k‐Leitlinie „Rationaler therapeutischer Einsatz von kaltem physikalischem
  - Plasma“
  - WHO‐Leitlinie „Antimikrobielle Resistenzen“
  - NICE‐Guideline „Pressure ulcers“
  - DIN SPEC 91315:2024‐08: Allgemeine Anforderungen an Plasmaquellen für die Erzeugung eines kalten Atmosphärendruckplasmas (CAP) für medizinische Anwendungen.
6. Für welchen Effekt bei der Behandlung von chronischen Wunden mit Kaltplasma liegt Evidenz aus klinischen Studien vor?
  - Verkürzte Hospitalisationsdauer um 50%
  - Signifikante Reduktion der bakteriellen Last und Verbesserung der Heilung
  - Vollständige Heilung innerhalb von 24 Stunden
  - Austausch konventioneller Verbände überflüssig
  - Ausschließlich kosmetische Verbesserung ohne Einfluss auf Heilung
7. Welche Nebenwirkung wurde bei Kaltplasma‐Behandlung von Wunden in Studien NICHT beobachtet?
  - Schwerwiegende unerwünschte Ereignisse (SAEs)
  - Leichte Erwärmung der Haut
  - Lokale Rötungen
  - Kurzzeitiges Kribbeln
  - Leichte Schmerzempfindung
8. Welche zusätzliche Wirkung konnte Kaltplasma in Leberverletzungsmodellen zeigen?
  - Verlangsamung der Koagulation
  - Starke hämostatische Wirkung durch Thrombozytenaktivierung
  - Hemmung der Thrombozytenaggregation
  - Verstärkte Blutungen durch Gefäßerweiterung
  - Induktion von Fibrinolyse
9. Welche Aussage zur klinischen Evidenz von Kaltplasma trifft zu?
  - Nur Tiermodelle zeigen Wirksamkeit
  - Es gibt keine prospektiven, randomisierten Studien
  - Mehrere RCTs belegen antimikrobielle und wundheilungsfördernde Effekte
  - Es ist ausschließlich als experimentelles Verfahren ohne Zulassung verfügbar
  - Die Wirkung ist nur bei chirurgischen Wunden nachweisbar
10. Welcher Mechanismus trägt nach Studien wesentlich zur durch Kaltplasma geförderten Mikrozirkulation bei?
  - Induktion einer lokalen Hypoxie
  - Erhöhung der endogenen NO‐Produktion und Vasodilatation
  - Blockade der Angiogenese
  - Hemmung der Leukozytenmigration
  - Absenkung der Gewebetemperatur

Liebe Leserinnen und Leser, der Einsendeschluss an die DDA für diese Ausgabe ist der 30. April 2026.

Die richtige Lösung zum Thema Unerwünschte Wirkungen von Januskinase‐Inhibitoren mit Relevanz für den dermatologischen Klinik‐ und Praxisalltag in Heft 09/2025 ist: 1b, 2d, 3c, 4c, 5a, 6e, 7d, 8e, 9d, 10c.

Bitte verwenden Sie für Ihre Einsendung das aktuelle Formblatt auf der folgenden Seite oder aber geben Sie Ihre Lösung online unter http://jddg.akademie-dda.de ein.
